# The Effect of Acute Hypohydration on Indicators of Glycemic Regulation, Appetite, Metabolism and Stress: A Systematic Review and Meta-Analysis

**DOI:** 10.3390/nu12092526

**Published:** 2020-08-20

**Authors:** Mitchell E. Zaplatosch, William M. Adams

**Affiliations:** Hydration, Environment and Thermal Stress Lab, Department of Kinesiology, University of North Carolina at Greensboro, Greensboro, NC 27412, USA; mezaplat@uncg.edu

**Keywords:** hydration, hypohydration, health, appetite, glycemic, metabolism

## Abstract

Evidence synthesizing the effects of acute body water losses on various markers of glycemic regulation, appetite, metabolism, and stress is lacking. Thus, the purpose of this review was to summarize the response of various hormonal changes involved in these physiologic functions to dehydration. A comprehensive literature search for peer-reviewed research in the databases PubMed, Scopus, CINAHL, and SportDiscus was conducted. Studies were included if they contained samples of adults (>18 years) and experimentally induced dehydration as measured by acute body mass loss. Twenty-one articles were eligible for inclusion. Findings suggested cortisol is significantly elevated with hypohydration (standard mean difference [SMD] = 1.12, 95% CI [0.583, 1.67], *p* < 0.0001). Testosterone was significantly lower in studies where hypohydration was accompanied by caloric restriction (SMD= −1.04, 95% CI [−1.93, −0.14], *p* = 0.02), however, there were no changes in testosterone in studies examining hypohydration alone (SMD = −0.17, 95% CI [−0.51 0.16], *p* = 0.30). Insulin and ghrelin were unaffected by acute total body water losses. Acute hypohydration increases markers of catabolism but has a negligible effect on markers of glycemic regulation, appetite, anabolism and stress. Given the brevity of existing research, further research is needed to determine the impact of hydration on glucagon, leptin, peptide YY and the subsequent outcomes relevant to both health and performance.

## 1. Introduction

Maintaining an adequate state of hydration is vital for optimizing human health and performance. Previous literature assessing the role of hydration on exercise performance has demonstrated the detrimental impact of acute hypohydration on aerobic [1,2,3] and anaerobic [4,5,6,7] exercise performance, as well as cognitive function [8,9,10,11]. In recent years, the focus has shifted from the short-term impact of hypohydration on exercise and cognitive performance to the role that inadequate fluid intake, also termed underhydration [12], has on health-related outcomes such as obesity [3,13,14,15,16,17,18], diabetes [19,20,21], and chronic kidney disease [22,23]. However, the existing evidence examining the associations between underhydration on the aforementioned health-related outcomes in humans has primarily come from retrospective analyses of population-based cohort studies [22]. Given the brevity of current literature investigating the independent effects of habitual water consumption on long-term health outcomes, acute-term (i.e., hours to days) studies manipulating body water have been able to investigate the role of body water loss on changes in various hormones that contribute to an individual’s long-term risk profile. Alterations in endocrine function, notably, changes in glycemic regulation, appetite control, metabolism, and stress, directly or indirectly influence several processes required for training adaptation [24,25] and can also impact one’s health status [26,27].

### 1.1. Glycemic Regulation

Recent evidence has suggested a link between body water deficits and impairments in blood glucose regulation, particularly in individuals with type 2 diabetics, attributed to elevations in the fluid regulatory hormone arginine vasopressin (AVP) [19,21,28,29,30]. Centrally, AVP binds to V1b receptors in the anterior pituitary gland [31] and through the secretion of cortisol, via the AVP-mediated activation of adrenocorticotropic hormone (ACTH), increases hepatic glucose output [32]. AVP also binds to V1a receptors in the liver to stimulate glycogen phosphorylase activity and thereby increase glycogenolysis [33]. Chronic elevations in blood glucose could lead to insulin resistance and subsequently the development of type 2 diabetes. Type 2 diabetes contributes to numerous macrovascular complications such as cardiovascular disease, stroke, and peripheral artery disease, as well as microvascular complications such as neuropathy, nephropathy, and retinopathy [34]. Chronic derangements in glycemic control could also pose a challenge for subsequent exercise. Elevations in cortisol and glucagon would reduce the availability of stored glycogen normally used for high intensity activity [35]. However, the magnitude of these changes and what level of hypohydration is required to cause these alterations in glucose metabolism in healthy individuals, both at rest and after activity, is not clear.

### 1.2. Appetite

The effect of manipulating hydration status on energy intake remains controversial but bears consideration for those seeking to improve their health through weight control. This factor is also important for individuals seeking to refuel following an exercise bout to ensure optimal recovery. Hypohydration may also influence the concentration of key hormones involved in energy intake, including reductions in the orexigenic hormone ghrelin [36] and has mixed effects on the anorexigenic hormones leptin and peptide YY (PYY) [37,38,39]. Sufficient nutrient intake is essential to maximize subsequent exercise performance and to support training adaptations through replenishment of glycogen stores [40] and promotion of muscle protein synthesis [41]. Conversely, for someone seeking weight reduction, increasing water intake is often recommended with the rationale this could decrease energy intake at a meal and decrease hunger [42,43]. While chronic underhydration has been associated with obesity [22], other reports suggest hypohydration reduces subsequent food intake [37]. Given the continuing rise in the prevalence of obese individuals and the associated health outcomes [44], research addressing potential contributors to weight gain may help alleviate this major public health problem.

### 1.3. Stress

Another biomarker, salivary marker alpha-amylase, has been associated with cardiometabolic risk [45]. In addition to its exocrine role of starch digestion, this molecule exerts effects on the endocrine system including alterations in pre-absorptive metabolic signaling and plasma glucose response to starches. Salivary alpha-amylase is commonly measured in conjunction with salivary cortisol as an indicator of sympathetic stress [46]. While this marker is also used as a measure of psychobiological stress [47], salivary alpha-amylase has also been assessed under states of physiological stress such as exercise and found to be predictive of increases in plasma norepinephrine [48]. Recent research has suggested hypohydration may alter salivary proteins through decreases in saliva flow rate, including alterations in salivary alpha amylase [49]. However, these findings are not consistent across studies. Additional factors such as the exercise intensity used to induce dehydration may have led to this variation [50]. Determining changes in this marker in response to hypohydration could determine the utility of this measure as a non-invasive marker of hydration status.

### 1.4. Metabolism

Reductions in total body water may also influence metabolism. Specifically, testosterone and cortisol are commonly used as indicators of anabolism and catabolism, respectively. The ratio of these hormones may be important for training adaptations [51]. Some studies have suggested hypohydration may exacerbate the catabolic response imposed by stressors such as exercise and heat stress [52,53]. Hypercortisolemia also contributes to suppressed immune function [54] and is linked with indicators of metabolic syndrome including obesity, type 2 diabetes, and hypertension [55]. However, the effects on this hormone are not consistent across intensities [53,56] or when subjects underwent a dehydration protocol on several separate occasions [57]. Thus, the magnitude of cortisol changes in response to hypohydration, as well as whether these alterations in hormonal balance are clinically versus statistically meaningful, is unclear. 

### 1.5. Mode of Dehydration

The methods used to induce dehydration can also influence these hormonal responses. Additional stressor commonly imposed in the dehydration literature include exercise, heat exposure, and caloric restriction, which can all independently influence hormonal levels [58,59,60]. With dehydration techniques that increase body temperature, the effects of hypohydration alone cannot be determined without sufficient time for cooling [6]. Other dehydration methods such as exercise protocols present their own physiological challenges, including changes in substrate availability, which contributes to hormonal perturbations. Differentiating between changes in these hormones due to the nature of the dehydration protocol versus the fluid loss itself is important to further explain the relationship between hypohydration and health.

Inconsistencies in current scientific literature related to the responses of hormones involved in glycemic regulation, appetite, metabolism and stress led to the development of this systematic review of the literature to determine the general trend of these responses. Variable responses have been shown in studies assessing ghrelin, perhaps related to the modality and proximity of the dehydration protocol to hormone assessment. For other hormones such as cortisol, the magnitude of change in response to reduced total body water yields a wide range of responses. Thus, the purpose of this review was to examine the influence of reductions in total body water on key hormonal indicators of, glycemic regulation (insulin, glucagon), appetite (ghrelin, leptin, PYY), metabolism (cortisol, testosterone) and stress (salivary alpha-amylase). Given the large variability in methods commonly used in hydration literature, this review also seeks to identify and address some of the key differences in these responses between the various methods used to induce fluid loss. Recognizing the impact of these acute changes in total body water on these markers is important to prompt the development of strategies to maintain fluid balance and reduce the risk of associated health outcomes.

## 2. Methods

This review is reported in accordance with the Preferred Reporting Items for Systematic Reviews and Meta-Analysis (PRISMA, File S1) statement [61]. This review’s methodology was previously specified and registered (PROSPERO registration number: CRD42020185392).

### 2.1. Eligibility Criteria

Articles were identified that investigated the relationship between hydration status and changes in select biomarkers of glycemic regulation (insulin, glucagon), appetite (ghrelin, leptin, PYY), metabolism (cortisol, testosterone), and stress (alpha-amylase). Studies were included if the research was conducted on humans; contained samples of adults (>18 years); experimentally induced dehydration by either fluid restriction, diuretic, heat exposure, exercise, or the combination of exercise and heat exposure. Studies were excluded if they were review articles, abstracts, theses or dissertations, not performed on humans, or contained subjects with pre-existing medical conditions (i.e., diabetes, CKD, hypertension). Acute change in body mass was selected as the indicator of hydration status, under the assumption that short term fluctuations in body mass primarily being attributed primarily body water losses, and not accounting for respiratory water losses or metabolic water release [62].

### 2.2. Search Strategy

A comprehensive search of the scientific literature was conducted in March 2020 using the electronic databases PubMed, Scopus, CINAHL, and SportDiscus. Searches included the following terms: (hydrat* OR euhydrat* OR dehydrat* OR hypohydrat* OR “fluid intake” OR “water intake” OR “fluid balance” OR underhydration OR “water consumption”) AND (leptin OR ghrelin OR PYY OR cortisol OR “alpha amylase” OR testosterone OR insulin OR glucagon). No restrictions were placed on the database searches, and no date range was specified, although “Humans” was specified in the PubMed database. Manual cross-referencing of retrieved included articles was conducted to identify any additional relevant articles.

### 2.3. Screening and Data Extraction

All articles were screened using the inclusion and exclusion criteria by the primary author (MEZ). Articles were cross-referenced between databases with duplicate entries removed. Studies deemed eligible based on title were then screened for abstract, and if still meeting eligibility criteria, the full text was analyzed. Data was extracted from each article in Microsoft Excel (Microsoft, Redmond, Washington, DC, USA), including author and year of publication, participant characteristics, study design (including method used to reduce total body water and level of body mass reduction), intervention, and outcomes (leptin, ghrelin, insulin, glucagon, testosterone, cortisol, salivary alpha amylase). Primary author (MEZ) checked the data extraction tool and discussed any uncertainties with last author (WMA).

### 2.4. Quality Assessment

Study quality was assessed using the Quality Assessment Tool for Quantitative Studies from the Effective Public Health Project, which provides a criterion by which to evaluate studies of different designs and rates studies as “strong”, “moderate”, or “weak” [63]. The quality domains addressed with this tool include selection bias, study design, confounders, blinding data collection method, withdrawals/dropouts. Quality domains for each article meeting inclusion criteria were rated by both authors. Any differences were discussed and consensus for final overall study rating agreed upon. Inter-rater agreement for the rating of methodological quality between included articles was computed using Cohen’s Kappa as k = 0.952.

### 2.5. Data Synthesis

Characteristics of the studies were summarized by participant characteristics, method used to achieve total body water loss, hormone or biomarker assessed, absolute change in hormone or biomarker, relative change in hormone or biomarker, and degree of change in each biomarker for every percent decrease in total body water.

### 2.6. Data Analysis

From each study, the number of subjects, means and standard deviations for hormones of interest was entered into RevMan Software Version 5.4 (Copenhagen: The Nordic Cochrane Centre, The Cochrane Collaboration, 2020) to calculate weighted mean differences and 95% confidence intervals (CI) using random-effects models [64] For each study, mean concentrations (hematologic or salivary) of the specific biomarkers of interest under conditions of greater reductions in total body water were compared against euhydration (normal body water). For hormone concentrations assessed using different measures (i.e., salivary versus hematologic), standardized mean differences were used for comparison. For studies assessing participants under separate conditions with different levels of fluid loss, comparisons were included between euhydration and each level of body mass loss. For example, in a study comparing three experimental trials measuring euhydration, 2.5%, and 5% body mass loss, comparisons were made between euhydrated and 2.5% BML and euhydrated and 5% BML. For studies assessing the effect of progressive dehydration within the same day, mean comparisons were only made between the baseline euhydrated state and the highest level of body water loss achieved. If unavailable within the manuscript, individual authors were contacted for raw means and standard deviations for the hormone concentrations and bodyweight measures. If either of these parameters were unavailable, the study was excluded from analysis. Study heterogeneity was determined by I^2^ statistic, where > 50% indicated significant heterogeneity between studies. All data were converted to SI units if not reported as such in their respective articles.

## 3. Results

### 3.1. Study Selection

**Figure 1:**
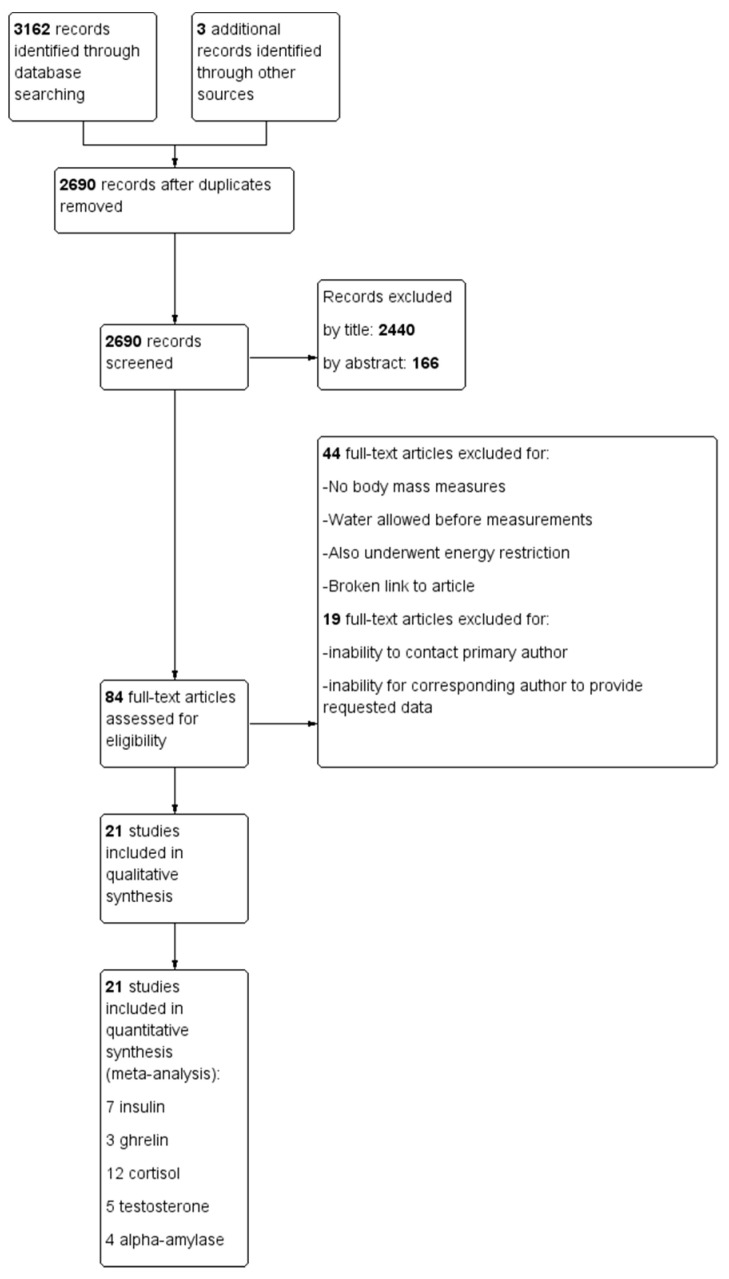
Flow chart of study selection.

Searches from all databases yielded 3162 articles. Three additional articles were added from a manual search of the reference lists of retrieved papers and during data acquisition, leading to 3165 total articles, from which 472 duplicates were removed, leaving 2690 assessed for eligibility. After screening by title/abstract, 2606 articles were removed, leaving 84 articles for the full-text screening. Forty-four full text articles were removed for methodological concerns including no body mass change measures (*n* = 17), allowing water or other fluids before trials (*n* = 18), participants primarily undergoing energy restriction rather than acute dehydration (*n* = 4), outside of age range (*n* = 1), case study (*n* = 1), arrived dehydrated (*n* = 1), microgravity (*n* = 1) as well as an inability to access two articles. Nineteen studies were removed due to inability to contact the corresponding author or inability to access raw data means and standard deviations [24,37,52,53,56,65,66,67,68,69,70,71,72,73,74,75,76,77,78,79]. Twenty-one articles [24,30,36,49,65,80,81,82,83,84,85,86,87,88,89,90,91,92,93,94,95] met all inclusion criteria and were included in this review (Figure 1). Among these studies, seven measured hormones involved in glycemic regulation [24,30,36,82,83,89,90], three measured appetite hormones [36,80,83], and fifteen measured biomarkers for metabolism and stress [30,49,57,81,84,85,86,87,88,90,91,92,93,94,95].

### 3.2. Study Characteristics

Characteristics of included studies are presented in Table 1. To reduce total body water, eight studies utilized heat exposure [30,80,81,83,85,86,90,93], fourteen used exercise [36,49,57,80,81,84,85,86,87,88,90,93,94,95], six used a combination of exercise and thermal exposure [80,81,85,86,90,93], seven used fluid restriction/low fluid prescription [30,36,57,80,81,83,93], two used a combination of food and fluid restriction [57,81] and one used diuretics [89]. On average, studies achieved a total body water loss of 2.45%.

### 3.3. Quality Assessment

Table 1 also lists the quality rating for each study using the Quality Assessment Tool for Quantitative Studies. No studies received a “strong” rating. Of the included studies, five were rated as “moderate” [36,49,84,86,87]. The remaining 16 studies received a “weak” rating. The resulting “weak” ratings were primarily due to the study design since few studies were true randomized clinical trials. Blinding has rarely been used in hydration literature; therefore, this domain was also scored lower among the included studies. Reporting of dropouts was another domain where most studies received a “weak” rating due to a failure to report these values. Given the large number of studies with a “weak” rating only as a result of lower scores in these three domains, the decision was made to include all of the studies in the initial analysis with later consideration for other influential factors such as mode of dehydration.

### 3.4. Effect of Hypohydration on Selected Hormonal Responses

Below we present the impact of reduction on total body water on each hormone or biomarker selected with meta-analyses where possible.

#### 3.4.1. Effect of Hypohydration on Hormones Involved in Glycemic Regulation

**Figure 2:**
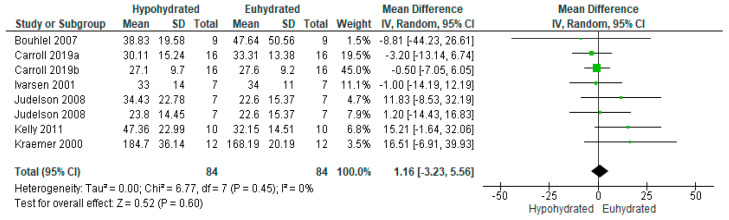
Forest plot of comparison for the weighted random effects meta-analysis of hypohydration on plasma insulin concentration (pmol/L). Data presented as standard mean difference with 95% confidence intervals comparing euhydrated to hypohydrated.

Seven studies [24,30,36,82,83,89,90] examined the relationship between reductions in total body water and hormones involved in glycemic regulation. Among the included studies, seven measured insulin [24,30,36,82,83,89,90] while only one included study measured glucagon concentrations [89]. Figure 2 illustrates the weighted mean difference for insulin between the greater fluid loss condition versus euhydrated conditions. Overall, there was no significant difference in insulin concentrations between participants when they were hypohydrated versus euhydrated (*n* = 84; MD = 1.16, 95% CI [−3.23,5.56], *p* = 0.60). There was high homogeneity among studies (I^2^ = 0%, *p* = 0.45). This finding was consistent regardless of the inclusion of studies assessing the post-prandial insulin response [30,83,89] versus fasting insulin levels [24,82,90]. On average, there was a 2.34% body mass loss in studies assessing insulin, with a 1.07 pmol/L increase in insulin for every 1% dehydration.

Only one study included in this review examined change in glucagon levels [89]. This study found a significant increase in plasma glucagon levels (*p* < 0.005) during alanine infusion after participants underwent diuretic-induced hypohydration of ~1% body mass loss, which resulted in a 32% increase in glucagon levels (28 pg/mL). However, this study did not isolate the independent effect of hypohydration versus the furosemide administration itself on glucagon concentration [96]. Regardless, this increase is substantial considering a reference range of 50–100 pg/mL, which could move an individual with levels at the higher end of this range out of the physiological norm.

#### 3.4.2. Effect of Hypohydration on Appetite Regulatory Hormones

**Figure 3:**
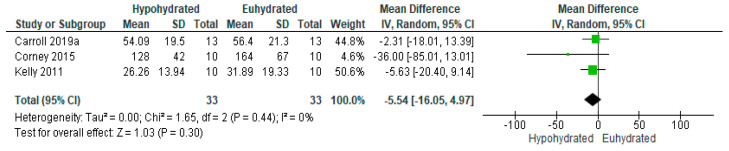
Forest plot of comparison for the weighted random effects meta-analysis of hypohydration on plasma ghrelin (pmol/L). Data presented using as mean difference with 95% confidence intervals comparing euhydrated to hypohydrated.

Three studies [36,80,83] focused on the effect of reductions in total body water on changes in appetite regulatory hormones. All three included ghrelin as an outcome measure, with two measuring total ghrelin [36,83] and one measuring acylated ghrelin [80]. The meta-analysis for ghrelin (Figure 3) revealed a non-significant effect of reductions in total body water on concentrations of this hormone (*n* = 33; MD = −5.54, 95% CI [−16.05, 4.97], *p* = 0.30). Collectively, these studies produced a mean 1.90% loss of total body water, yielding a 13.87 pmol/L decrease in ghrelin for every 1% body mass lost.

Only one study with raw data available from the authors examined changes in leptin and PYY [36], and the effect of hypohydration on each of these satiety hormones was nonsignificant (Table 1).

#### 3.4.3. Effect of Hypohydration on Markers of Metabolism and Stress

Sixteen studies examined the effect of hypohydration on markers of metabolism and stress. Among these studies, thirteen studies [30,49,57,81,84,85,87,88,90,92,93,94,95] examined the relationship between reductions in total body water and changes in cortisol, six studies measured testosterone [57,81,90,91,92,95], and four measured salivary alpha-amylase [49,87,92,94].

**Figure 4:**
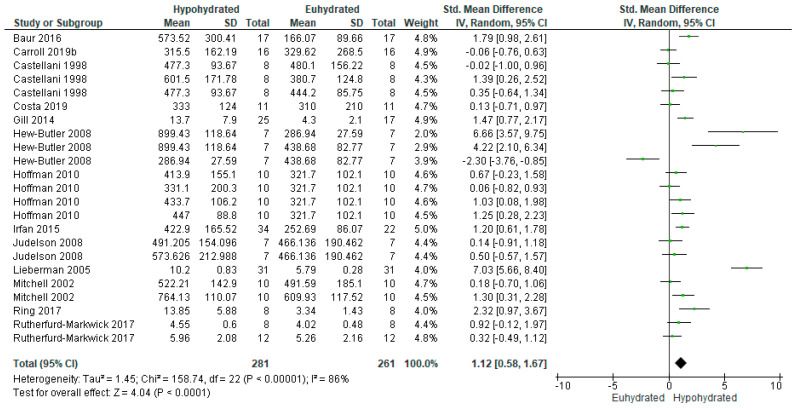
Forest plot of comparison for the weighted random effects meta-analysis of hypohydration on plasma cortisol concentration (nmol/L). Data presented as standard mean difference with 95% confidence intervals comparing euhydrated to hypohydrated.

Figure 4 illustrates the effect of hypohydration on cortisol response. Nine studies [30,57,80,81,84,85,88,90,93] measured serum cortisol, while 4 measured salivary cortisol [49,87,92,94]. Overall, there was a significant increase in cortisol levels with hypohydration (*n* = 281 vs 261; SMD = 1.12, 95% CI [0.58, 1.67], *p* < 0.0001). However, there was high heterogeneity among these studies (I^2^ = 86%, *p* < 0.00001). On average, there was a mean 2.85% dehydration among comparisons measuring serum cortisol, with a 43.81 nmol/L increase in cortisol for every 1% increase in total body mass loss. For the studies measuring salivary cortisol, there was a mean 2.10% dehydration, with a 2.72 nmol/L increase in cortisol for every 1% increase in total body mass loss.

**Figure 5:**
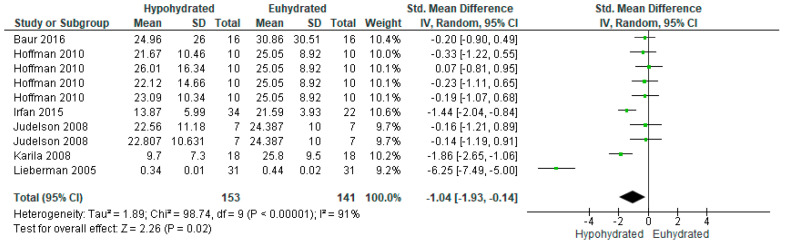
Forest plot of comparison for the weighted random effects meta-analysis of hypohydration on plasma testosterone concentration (nmol/L). Data presented as standard mean difference with 95% confidence intervals comparing euhydrated to hypohydrated.

**Figure 6:**
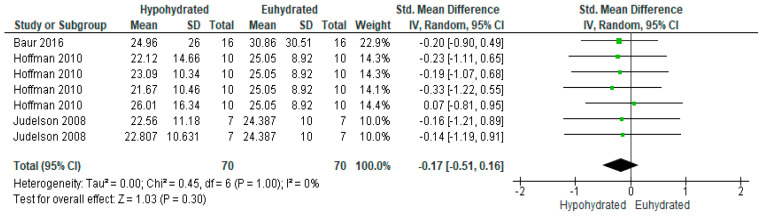
Forest plot of comparison for the weighted random effects meta-analysis of hypohydration on plasma testosterone concentration (nmol/L) without the inclusion of studies also incorporating food restriction. Data presented as standard mean difference with 95% confidence intervals comparing euhydrated to hypohydrated.

Among the five studies with available data to compare testosterone, there were 10 comparisons between euhydrated and hypohydrated (Figure 5). Hypohydration significantly decreased serum testosterone levels (*n* = 153 vs 141; SMD = −1.04, 95% CI [−1.93, −0.14], *p* = 0.02). There was significant heterogeneity among studies (I^2^ = 91%, *p* < 0.0001). This heterogeneity likely occurred due to the addition of caloric restriction in some of the studies, which has been shown to independently decrease testosterone levels. When conducting a subgroup analysis (Figure 6) removing the studies which incorporated a combination of food and fluid restriction [81,91,92], the effect was no longer significant (*n* = 70; SMD = −0.17, 95% CI [−0.51, 0.16], *p* = 0.3). Including all studies, on average, there was a mean 3.44% dehydration among studies analyzed, with a 1.15 nmol/L decrease in testosterone for every 1% increase in total body mass loss before subgroup analysis. After removing the studies also incorporating food and fluid restriction, there was a mean 2.8% dehydration among studies, with a 0.68 nmol/L decrease in testosterone for every 1% increase in total body mass loss.

**Figure 7:**
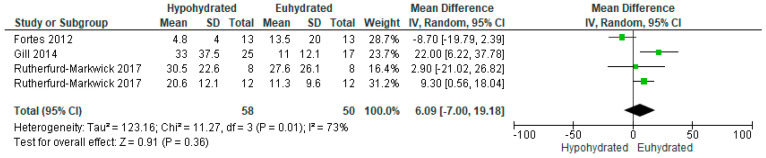
Forest plot of comparison for the weighted random effects meta-analysis of hypohydration on salivary alpha amylase secretion (U/mL). Data presented as mean difference with 95% confidence intervals comparing euhydrated to hypohydrated.

Salivary alpha-amylase was analyzed in four studies, reported as a variety of metrics including secretion rate [86,87,94], concentration [49,87], and activity [86,94]. Since the interpretation of these metrics can vary slightly based on the measure used [97] and given the limited number of studies which have assessed the response of these markers to acute body water deficits, all were included in the analysis. As expressed in Figure 7, there was no significant effect of reductions in total body water on salivary alpha-amylase secretion rate (*n* = 58 vs 50; MD = 6.09, 95% CI [−7.00, 19.18], *p* = 0.36). There was significant heterogeneity among studies (*p* = 0.01, I^2^ = 73%). On average, there was a mean 1.38% dehydration among studies analyzing secretion rate, with a 9.28 U/mL increase in salivary alpha-amylase secretion rate for every 1% increase in total body mass loss.

**Figure 8:**
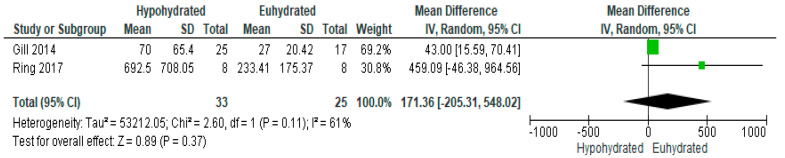
Forest plot of comparison for the weighted fixed effects meta-analysis of hypohydration on salivary alpha-amylase concentration (U/mL). Data presented as mean difference with 95% confidence intervals comparing euhydrated to hypohydrated.

For the two studies which measured salivary alpha-amylase concentration (Figure 8), there was a non-significant increase in the concentration of this biomarker when hypohydrated compared to euhydration (*p* = 0.37, MD = 171.36, 95% C.I. [−205.31, 548.02]. However, there was moderate heterogeneity among studies (*p* = 0.11, I^2^ = 61%), with a greater increase in salivary alpha-amylase in the study with a greater level of dehydration (3.01% in Ring et al. vs 1.6% in Gill et al.).

**Figure 9:**
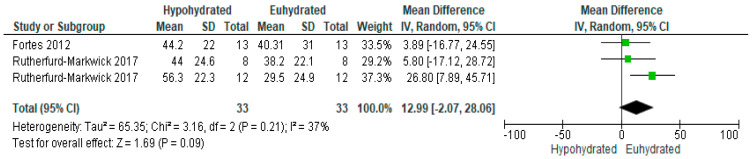
Forest plot of comparison for the weighted random effects meta-analysis of hypohydration on salivary alpha-amylase activity (U/mL). Data presented as mean difference with 95% confidence intervals comparing euhydrated to hypohydrated.

Two studies examined salivary alpha-amylase activity. As expressed in Figure 9, there was a non-significant increase in salivary alpha-amylase activity in the hypohydrated state compared to euhydrated (*p* = 0.09, MD = 12.99, 95% C.I. [−2.07, 28.06]. There was small heterogeneity among studies used for this comparison (*p* = 0.21, I^2^ = 37%).

## 4. Discussion

The purpose of this systematic review and meta-analysis was to summarize the influence of dehydration as measured by acute body mass loss on hormonal indices of glycemic regulation, appetite, metabolism, and stress. Results from the studies suggest acute reductions in total body water significantly increase cortisol and, when combined with caloric restriction, decrease testosterone concentrations. Acute reductions in total body water did not affect hormones involved in glycemic regulation or appetite control.

### 4.1. Glycemic Regulation

Our findings suggest that acute body water deficits do not impact circulating levels of insulin but may increase glucagon. These findings are similar to a study by Jansen et al., [98] which found that inducing a hyperosmotic state via infusion of hypertonic saline resulted in a greater area under the curve for glucagon and significantly elevated glucose concentration at minutes 60 and 90 of an oral glucose tolerance test, while insulin was unaffected. Furthermore, substantially increasing one’s water intake seems to both lower serum copeptin, a surrogate marker for arginine vasopressin (AVP), and decrease glucagon levels among “water responders”, though insulin and glucose levels remain similar [19]. Therefore, it may be that these changes in glycemic regulation depend upon one’s habitual water intake and, perhaps, one’s basal copeptin/AVP levels. In animal models, when exogenous AVP was given, results show increases in both insulin and glucagon in response to V1b receptor binding in the liver, but the threshold for increased insulin appears to be higher than glucagon [33]. Perhaps the changes in AVP were not significant enough in the included studies to alter circulating insulin concentrations in humans, although only one study [30] measured copeptin. Carroll et al. [30] observed a significant increase in copeptin but no change in glycemic control. Unfortunately, glucagon was not included in our meta-analysis, but the one study included in our review which assessed this hormone following alanine infusion did find a significant increase in plasma glucagon, whereas insulin remained similar between euhydration and hypohydration achieved via furosemide administration [89]. Since furosemide administration itself can impact glucagon concentrations following arginine administration [96], these findings warrant further investigation to separate the effect of body water losses from other potential cofounders such as downstream effects induced by diuretic use.

Characterizing the effects of manipulating fluid intake on insulin and glucagon should be further explored to better understand the role hydration plays in glycemic regulation. If favorable changes in these hormones can be achieved by preventing fluid losses in total body water, this could reduce the need for alternative, expensive strategies for glycemic control such as medications, and could be included with dietary and physical activity recommendations for proper blood glucose management. There remains uncertainty regarding the role of hydration on glucose regulation [19,28,29,99,100] and the precise mechanism by which this occurs. Specifically, the differential responses observed in populations with diabetes versus those without diabetes may have been due to the glucosuria observed in individuals with diabetes who went off of their medication for the study [100]. Regardless, based on our results it would seem any impact of reductions in total body water on glucose regulation is not insulin mediated.

### 4.2. Appetite

In our analysis, there was no effect of reductions in total body water on ghrelin levels (Figure 3). This analysis pooled the results of studies [36,80,83] looking at both fasting ghrelin levels and ghrelin response to a meal. In another study for which raw data was inaccessible for inclusion in the present review [37], eight men underwent exercise induced hypohydration before consuming cereal bars ad libitum either hypohydrated or following fluid two hours of fluid replenishment. Energy intake was significantly reduced in this study by ~700 kcals. Although ghrelin, leptin, and PYY were similar before the meal, after eating PYY was significantly increased under the rehydration condition due to the increased caloric intake. Exercise at a moderate intensity has been shown to independently reduce relative energy intake [101]. Thus, these differences in appetite regulatory hormones may be influenced by either the inclusion of or proximity of exercise and hypohydration in relation to food intake. In our analysis, the studies by Kelly et al. [36] and Corney et al. [80] also included exercise to induce hypohydration, but the closer proximity of exercise to ghrelin measurement for Kelly et al. may have contributed to the significant reduction in ghrelin observed in this study. It is also important to note that two out of the three included studies [36,80] assessed acetylated ghrelin as compared to total ghrelin in one study [83]. Acylated ghrelin has been shown to exert additional actions including increased circulating growth hormone, ACTH, and cortisol [24]. Additional research is needed in this area to capture differences in fasting ghrelin versus post-prandial total and acylated ghrelin in response to various states of hydration, with or without exercise.

Although not captured in the present review, there are several additional hormones involved in appetite regulation. Notably, anorexigenic hormones glucagon-like peptide 1 (GLP-1) and cholecystokinin (CCK) produced in the gut, as well as orexigenic hormones agouti-related peptide (AGRP), and (NPY) produced from neurons in the arcuate nucleus of the hypothalamus, have not been explored in humans with regards to changes in hydration status [27]. However, changes in these hormones in response to dehydration have been linked with dehydration induced anorexia in rats [27]. The arcuate nucleus also expresses pro-opiomelanocortin (POMC), which acts as a precursor for several regulatory molecules, including ACTH (see Section 4.3). Since ACTH has been shown to change in response to dehydration in humans, future studies should examine how both the neural and subsequent hormonal factors involved in appetite regulation respond to dehydration.

Additional studies should also examine the effect of chronic underhydration from habitual low fluid intake on appetite regulatory hormones as compared to acute reductions in total body water. This will help expand our understanding of the complex relationship between hydration, energy intake, and obesity.

### 4.3. Metabolism and Stress

Based on our analysis, various methods used to acutely induce total body water losses significantly increased plasma cortisol levels (Figure 4). However, it is known that exercise also independently impacts the cortisol response, with longer duration or higher intensity activities tending to further exacerbate the cortisol response [59,102,103]. All but one of the studies included in the review [49,57,81,84,85,87,90,92,93,95] utilized exercise of varying intensities to induce fluid loss, which may prohibit our understanding of the independent influence of hypohydration on cortisol secretion. For instance, although the study by Hew-Butler et al. [88] produced greater dehydration during 1 h of steady-state exercise, the shorter duration but higher intensity VO_2_ max trial induced a greater cortisol response. Carroll et al. [30] did not find any influence of hypohydration on cortisol when incorporating a combination of heat tent exposure and fluid restriction without an exercise component. This is despite evidence that heat exposure seems to also contribute to the rise in cortisol during exercise [77,104]. Thus, it may be that the effects of heat exposure on cortisol are dependent upon the co-occurrence of an additional stressor such as exercise. Given that exercise would exacerbate the rate of fluid loss compared to passive exposure, perhaps these stressors (heat and exercise) act synergistically to increase cortisol.

The proposed mechanism for increased cortisol with hypohydration has been explored and attributed to AVP-mediated stimulation of the conversion of corticotropin-releasing hormone to ACTH and subsequently increasing cortisol [100]. However, this response was not found in the study by Carroll et al. which examined ACTH and copeptin in addition to cortisol and found no significant increase in the latter despite increases in copeptin [30]. In contrast, current evidence has found higher cortisol levels among low drinkers (consuming < 1.2 L fluid/day) compared to high drinkers (2–4 L/day) [20]. Therefore, the influence of underhydration from chronic low intake versus acute hypohydration from exercise or heat exposure requires additional exploration.

When examining the influence of body water losses on anabolism, our findings showed that a decrease in total body water resulted in a decline in testosterone (Figure 5). However, upon removing studies that also included acute caloric restriction [81,91,92], the effect of fluid loss alone (either through exercise, heat exposure, a combination, or fluid restriction), did not have a significant effect on testosterone (Figure 5). Evidence suggests that, in the long term, caloric restriction is associated with reductions in serum total testosterone and free androgen index [105], particularly if combined with excessive exercise [106]. However, this decline seems reversible with a return to adequate energy intake [53]. Maresh et al. showed a reduced testosterone:cortisol ratio in cross-country athletes who completed a training session when hypohydrated by 5% of body mass [53]. Thus, hypohydration may lead to an unfavorable hormonal balance of anabolic and catabolic hormones which could impair training adaptations. It also seems these effects are more pronounced with higher levels of hypohydration, coinciding with the effect on performance decrements [59].

The influence of hypohydration on the stress response was not significant based on alpha-amylase secretion rate (Figure 7) and concentration (Figure 8), but activity was trending towards significance (Figure 9). This marker has been suggested to be representative of the sympathetic stress response [107]. Although to the authors’ knowledge there is no established norm for salivary amylase activity, the 33.8% increase when hypohydrated compared to euhydrated, while not statistically significant, may be worth further exploration. Some studies have found elevations in salivary alpha-amylase concentrations in individuals with diabetes [108,109]. However, it seems consideration for hydration status when assessing salivary alpha-amylase activity in past and future research may be worth consideration. Also, with the desire for rapid, non-invasive field assessments of hydration, additional research in the area of salivary markers is warranted. Repeated stress may adversely affect immune function [110] and thus predispose the athlete or general exerciser to illness. Thus, one strategy to prevent an excessive sympathetic stress response could be to ensure either maintenance of fluid balance throughout activity or rapid rehydration following activity. However, given the limited number of studies in the review assessing this marker and the high heterogeneity of study responses for both secretion rate and activity, additional study is warranted before definitive conclusions may be drawn for this marker.

Taken together, these results suggest decreases in total body water contribute to an unfavorable hormonal environment which may blunt anabolism if combined with additional perturbations such as reduced energy availability while increasing catabolism. Thus, ensuring adequate fluid consumption during activity as well as replacement following activity may help create a more favorable hormonal response to help adapt and prepare for subsequent activity.

### 4.4. Influence of the Method to Reduce Total Body Water

A major limitation in attempting to gather hydration literature for a meta-analysis is the variability among dehydration protocols. An attempt was made to complete subgroup analyses for the influence of each hormone on hypohydration. Unfortunately, such subgroup analysis either could not be conducted given a limited number of studies (salivary alpha-amylase, glucagon, leptin, PYY) or large variability within a type of fluid reduction strategy (i.e., highly variable exercise protocols used in studies assessing the change in cortisol with hypohydration). However, a subgroup analysis on the influence of hypohydration on testosterone was completed, which suggested the decrease in this hormone in the main analysis was likely skewed to the additional weight-reduction regimens practiced in these particular studies [57,81,91,92]. Additionally, true total body water losses are more likely to be less than estimated from body mass change alone due to increased water release from glycogen breakdown that occurs during more prolonged activity [111], which could have influenced the calculated magnitude of change for each hormone for each percentage of body mass lost. This was particularly evident in the cortisol response in our studies, where studies employing longer duration endurance events to induce dehydration [87,88,92] induced higher cortisol levels compared to shorter duration activities [94] or compared to those with hormonal responses measured the following day [90]. Given that these multiple additional factors coinciding with reductions in total body water may also influence hormonal responses, care should be taken to ensure appropriate isolation of body water changes if this is to be used as the predictor variable for the change in these biomarkers.

This review sought to examine the effect of hypohydration on hormonal indices compared to either a separated euhydrated condition or a pre-exercise baseline. However, it is worth noting that some studies also included a subsequent exercise bout in a hypohydrated state [90]. This review does not capture the hormonal responses to an exercise bout when initiated in a hydrated versus underhydrated state but rather the influence on these hormones during subsequent resting conditions. Determining the effect of beginning an exercise bout in a hypohydrated state on the subsequent hormonal response, and its implications for exercise recovery, is warranted.

### 4.5. Limitations

The hormone list included in this review is not all-inclusive. Additional research should be conducted to capture the full range of the effects changes in total body water have on endocrine function, including but not limited to the effects on growth hormone, IGF-1, and thyroid hormone. This can provide additional insights into the mechanisms by which changes in hydration status impact health and human performance. Also, the studies included in this review focused on the acute response to reductions in total body water. Further research should consider the effects of chronic underhydration on endocrine function in terms of glucose regulation, appetite, metabolism, and stress response.

Very few of the included studies recruited female participants (males: n = 272, females: n = 55). Although the cortisol response to exercise seems similar across menstrual cycle phases with prolonged exercise [112], the cortisol response to psychological stressors may vary [113]. Among studies looking at appetite regulation, some referred to the difficulty to control for the complex interactions between menstrual cycle hormones and appetite independent of the influence of menstrual cycle on fluid regulatory hormones. For example, both leptin and PYY have been shown to change significantly across the menstrual cycle [114,115], while changes in ghrelin seem to occur in females with menstrual cycle disturbances [116]. Thus, it is possible that changes in hormone levels in some of the studies included in this review with female participants could have been confounded by variable menstrual cycle stages among participants. Future research looking at hormonal changes with hypohydration should seek to recruit female participants in the same phase of their menstrual cycle and delve into the changes in these responses to losses in total body water across the menstrual cycle.

Lastly, several older papers [70,71,74] were found in the search that would have provided additional insight into these hormonal relationships, but their data was unavailable. In attempting to contact the corresponding authors for access to the raw data, some also noted difficulty acquiring such data due to current restrictions in place at different universities for accessing their campus during the COVID-19 pandemic [37,56].

The studies employed in this analysis which utilized fluid restriction attempted to create a total body water deficit that may be common among those who habitually under consume fluid. However, no experimental study has specifically addressed whether chronic low fluid intake (i.e., > 3 days) with a coordinated increase in vasopressin levels, termed underhydration [12], has a differential effect on hormonal responses compared to acute dehydration. Although the effect of increasing habitual water consumption on glucoregulatory hormones has been tested, the effect on the other hormones discussed in this review is less clear. As there remains no clear “gold standard” for hydration assessment [117], such studies should employ multiple methods to determine fluid balance, including serial body mass measures, 24 h urine collections, urine osmolality, urine specific gravity measurements [118] as well as blood biomarkers (i.e., vasopressin or its more stable surrogate marker copeptin and plasma osmolality).

## 5. Conclusions

To our knowledge, this is the first systematic review to examine hormonal responses to hypohydration concerning glycemic regulation, appetite, metabolism, and stress. In summary, our meta-analytic findings revealed reductions in total body water increase plasma cortisol levels but decrease plasma testosterone levels. This change in the hormonal milieu may acutely impede training adaptations and thereby impact later performance. These changes could also contribute to adverse health outcomes resulting from impaired immunity. Conversely, reductions in total body water did not significantly impact hormones involved in glycemic regulation or appetite control. Given the limited number of studies available, more research is needed to examine the influence of acute reductions in total body water on glucagon as well as additional appetite regulatory hormones including leptin and PYY. Expanding our understanding of the role total body water balance plays in other physiological systems will help enhance current understanding of the role hydration plays in both adaptations to exercise and health.

## Figures and Tables

**Table 1:** Characteristics of the 21 studies included in review analyzing the influence of total body water loss on hormone concentrations.

Study	Comparison	Hormone/Biomarker	Participants	Age	Method to Reduce Total Body Water	% BML	Relative Hormone Change in Study	Absolute Hormone Change for Every 1% Increase in BML	Overall Outcome	Quality
Baur (2016)	Pre vs Post Ultraman Triathlon	cortisol, testosterone	18 (14 male, 4 female); cortisol (*n* = 17), testosterone (*n* = 16)	40 ± 7 years	Multi-stage triathlon	3.9	cortisol: +245.35%; testosterone: −19.12%	cortisol: +104.47 nmol/L; testosterone: −1.51 nmol/L	Significant increase in cortisol (*p* = 0.00005) and significant decrease in testosterone (*p* = 0.033) from pre-post race.	moderate
Bouhlel (2008)	Before Ramadan vs 2 weeks into Ramadan	insulin	9 young men from national rugby team	19 ± 2 years	Reduced food and fluid intake (3.7 L average to 2.6 L average)	2.24	insulin: −18.51%	insulin: −3.94 pmol/L	No significant difference in fasting insulin levels after 2 weeks of Ramadan.	weak
Carroll (2019)a	Euhydration vs Hypohydration	ghrelin, insulin	16 adults (8 female)	30 ± 9 years	Low water content foods, 1 h dehydration in heat tent, water prescribed (3 mL/kg body mass vs 40 mL/kg lean mass plus 150% sweat losses).	1.9 ± 1.2	ghrelin: −9.63%; insulin: −9.61%	ghrelin: −10.12 pg/mL; insulin: −1.69 pmol/L	Post-prandial ghrelin similar between Euhydration and Hypohydration (*p* = 0.736)	weak
Carroll (2019)b	Euhydrated vs Hypohydration	cortisol, insulin					cortisol: −4.28%; insulin: −1.71%	cortisol: -7.43 nmol/L; insulin: −0.27 pmol/L	No significant difference in insulin (*p* = 0.200)) during OGTT. No difference in plasma cortisol (trial x time *p* = 0.674).	weak
Castellani (1998)	Pre-DH and Post-DH for NF, ISO, HYPO	cortisol	8 males	22 ± 0.8 years	Alternating 25-min cycling and treadmill walking with 5-min rests between. CHO given prior to second piece of exercise to offset glycogen loss during DH.	4.1 ± 0.1	cortisol: NF +7.45%, ISO +0.6%, HYPO +60%	cortisol: NF +8.07 nmol/L, ISO + 0.68 nmol/L, HYPO +53.85 nmol/L	No significant difference in cortisol from pre- to post- dehydration under any condition (*p* > 0.05).	moderate
Corney (2015)	13 h Post Exercise HYPO vs RE	acylated ghrelin	10 males	24 ± 1.2 years	Evening exercise in 35° C and either rehydrate with 175% BML (RE) or given 200 mL water (HYPO).	2.78 ± 0.48	acylated ghrelin: −22.0%	acylated ghrelin: −36 pmol/mL	No main effect of trial (*p* = 0.124) or interaction effect (*p* = 0.318) on acylated ghrelin.	weak
Costa (2019)	Water vs no water during exercise	cortisol	11 competitive male endurance runners	34 ± 11 years	2-h running at 70% VO_2_ max in 24.7 ± 1.7 °C, 46 ± 9% RH.	3.1	cortisol: +7.42%	cortisol: +7.42 nmol/L	Insignificant increase in plasma cortisol pre- to post-exercise (*p* = 0.098).	moderate
Fortes (2012)	Progressive hypohydration with water vs without water	salivary alpha-amylase secretion rate	13 healthy adults (9 males, 4 females)	24 ± 5 years	Cycling at 55% Peak Power Output (152 ± 32 Watts) in 33C 50% RH	1, 2, 3	alpha-amylase secretion: −64.4%	alpha-amylase secretion: −2.0 U/mL	Significant decrease in SAA secretion rate at 3% BML (44%), *p* < 0.001; no effect on SAA activity (*p* = 0.89)	moderate
Gill (2014)	Ultra-endurance runners and controls	salivary alpha amylase concentration, salivary alpha-amylase Secretion, Salivary Cortisol	25 ultra-endurance runners (19 males, 6 females); 17 Control (6 male, 11 female)	39 ± 7 years ultra-endurance runners vs 32 ± 11 Years Control	Ultramarathon (122–208 km)	1.6±2.0	alpha-amylase concentration: +159.26%; alpha amylase secretion: +200%, cortisol: +218.61%	alpha-amylase concentration: +26.99 U/mL; alpha-amylase secretion: +13.75 U/mL, Cortisol: +5.88 nmol/L	Significantly increased salivary alpha-amylase secretion rate (*p* < 0.001) and Cortisol Responses (*p* < 0.001)	moderate
Hew-Butler (2008)	Post-exercise measures following all exercise bouts	cortisol	7 well-trained endurance runners (5 males, 2 females)	44 ± 4 years	Ultramarathon vs 60 min steady state run vs VO_2_ max test	Ultramarathon: 4±0.4 Steady state: 2.0 ± 0.1, VO2 max 0.30 ± 0.10	cortisol Ultramarathon vs steady state: +213.46%; Ultramarathon vs VO2 max: +105%; Steady state vs VO2 max: −34.59%	cortisol Ultramarathon vs steady state: +306.25 nmol/L; Ultramarathon vs VO2 max: +124.53 nmol/L; Steady state vs VO2 max: −89.26 nmol/L	Significantly higher cortisol following ultramarathon compared to steady state (*p* < 0.01) and VO2 max (*p* < 0.01)	weak
Hoffman (2010)	Hypohydration vs Baseline for W, DHY, LDAG, HDAG trials	cortisol, testosterone	10 active males	20.8 ± 0.6 years	Overnight food and fluid restriction to 1.03(1.3)% body mass loss. Then active dehydration protocol next morning: treadmill walking at 3.4 mi/h at 2% incline in training suit (long cotton heavy weight fleece sweat pants and top). 62.5 (44.2) min to reach weight loss.	2.5	cortisol HHY vs BL for DHY trial: +34.81%; HHY vs BL for W trial: +38.9%; HHY vs BL for LDAG: +28.66%; HHY vs BL for HDAG: +2.92% testosterone: HHY vs BL for DHY trial: −7.82%; HHY vs BL for W trial: −13.49%; HHY vs BL for LDAG: 3.83%; HHY vs BL for HDAG: 11.69%	cortisol: HHY vs BL for DHY trial: +34.81%; HHY vs BL for W trial: +38.9%; HHY vs BL for LDAG: +28.66%; HHY vs BL for HDAG: testosterone: HHY vs BL for DHY trial: 0.784 nmol/L; HHY vs BL for W trial:1.35 nmol/L; HHY vs BL for LDAG: −0.384 nmol/L; HHY vs BL for HDAG: 1.17 nmol/L	No significant differences in cortisol or testosterone after hypohydration.	weak
Irfan (2015)	Plasma osmolarity > 290 mOsm/L vs <290 mOsm/L	cortisol, testosterone	34 vs 22 elite male wrestlers	22.30 ± 2.43 years	Variable among wrestlers, including sauna, intense exercise and fluid restriction 1–5 days before competition	2.49	cortisol: + 67%; testosterone: −35.75%	cortisol: + 68.35 nmol/L; testosterone: −3.10 ng/dL	Significant increase in cortisol (*p* = 0.001) and significant decrease in testosterone (*p* = 0.001) for those participants considered dehydrated compared to euhydrated based on plasma osmolarity.	weak
Ivarsen (2001)	Euhydrated vs Hypohydration	glucagon, insulin	7 healthy males	23	Diuretic (furosemide)	1	glucagon: +23.52% insulin: −3.03%	glucagon: 36 pg/mL; insulin: +1 pmol/L	Significant increase in plasma glucagon concentration during alanine infusion (*p* < 005); no significant difference in insulin concentration	weak
Judelson (2008)	Euhydrated vs 2% Hypohydration; Euhydrated vs 5% Hypohydration pre-exercise	cortisol, insulin, testosterone	7 resistance trained males	23 ± 4 years	Combination fluid/fluid-rich food restriction, then returned and walked on treadmill 1.5 m/s 3% incline in environmental chamber 36–37C, 40–50% RH), repeated walking during all trials. Rehydrated with normal saline to achieve +0.5% over desired dehydration level to account for overnight water losses.	2.5, 5	cortisol EUH vs HY50: +23.05%; EUH vs HY25: +5.38%; testosterone EUH vs HY50: −7.49%; EUH vs HY25: −6.48%	cortisol EUH vs HY50: +23.37 nmol/L; EUH vs HY25: +11.40 nmol/L; testosterone EUH vs HY50: 0.83 nmol/L; EUH vs HY25: 0.34 nmol/L	HY50 cortisol significantly greater than EU before resistance exercise; pre-resistance exercise insulin levels significantly higher in HY50 vs EU; no significant difference in testosterone pre-exercise.	weak
Karila (2008)	Before versus After rapid weight reduction	testosterone	12 healthy male wrestlers	21.9 (17.8–31.7) years	Combination food and fluid‘ restriction by decreasing carbohydrate and fat intake in first 2–3 weeks, caloric restriction, then heavy exercise in hot sauna and fluid restriction.	8.2 ± 2.3 loss from all combined	testosterone: −63%	testosterone: −1.96 nmol/L	Significant decrease in serum testosterone.	weak
Kelly (2012)	Exercise DH vs Exercise HY Post Meal	ghrelin, leptin, PYY, insulin	10 healthy, active males	21.4 ± 1.3 years	Treadmill running for 45 min at 70% VO_2_ peak followed by fluid restriction.	2.3	ghrelin: −17.6%; leptin: 6.52% decrease; PYY: −3.92%;insulin: +45.2%	ghrelin: -18.56 pg/mL; leptin: -102.85 pg/mL; PYY: −3.59 pg/mL; insulin: +14.60 pmol/L	Significant decrease in ghrelin during DH compared with CON (*p* = 0.045) and HY trials (*p* = 0.014). No significant effect of hypohydration on leptin or PYY.	moderate
Kraemer (2001)	Baseline AM vs Pre-Match 1	insulin	12 male collegiate wrestlers	19.33 ± 1.16 years	Variable food and fluid restriction, exercise	3.89	insulin: −8.93%	insulin: −3.94 pmol/L	No significant difference in insulin (*p* > 0.05)	weak
Lieberman (2005)	Prefield Day 1 6 pm vs Postfield Day 4 6 pm	Salivary cortisol, salivary testosterone	31 male U.S. Army officers from an elite unit	31.6 ± 0.4 years	Simulated combat, food restriction, fluid restriction, sleep deprivation	5	cortisol: +76.16%, testosterone: −23.16%	cortisol: +0.88 nmol/L; testosterone: −0.02 nmol/L	Significant increase in salivary cortisol and significant decrease in salivary testosterone (*p* < 0.001)	weak
Mitchell (2002)	Euhydrated + Heat (EH) vs Hypohydrated + Heat (HH); Euhydrated Neutral (EN) vs Hypohydrated Neutral (HN)	cortisol	10 moderately trained males	24.7 ± 6.6 years	4 cycle ergometer rides at 55% VO_2_ peak either in a hot or neutral environment and either with or without fluid replacement throughout exercise	1, 2.4	cortisol: EH vs HH: +6.23%; EN vs HN +25.28%	cortisol: EH vs HH +30.62 nmol/L; EN vs HN +64.25 nmol/L	Significant increase in cortisol in hot environment regardless of hydration status. Cortisol significantly lower compared to pre-exercise in all conditions except after completing exercise hypohydrated in a hot environment (*p* < 0.05).	weak
Ring (2017)	Baseline vs Post Last Interval	salivary alpha-amylase concentration, salivary cortisol	10 males	25.5 ± 3.7 years	120 min of running in eight 15-min intervals with 8 min of rest between each	2.9	alpha-amylase: +197.69%; cortisol: +314.67%	alpha-amylase: +152.52 U/mL; cortisol: +3.5 nmol/L	Non-linear increase in salivary cortisol and salivary alpha amylase with progressive hypohydration.	moderate
Rutherfurd-Markwick (2017)	Control vs Exercise	salivary alpha amylase activity; salivary alpha amylase secretion rate; salivary cortisol	20 active adults (8 males, 12 females)	27.4 ± 5.9 years	Moderate intensity cycling (70% peak power) for 60 min	0.23 (males), 0.70 (females)	alpha amylase activity males: +15.18%; alpha amylase activity females: +90.85%; alpha amylase secretion males: +10.51%; alpha amylase secretion females: +82.3%	alpha amylase activity males: +25.78 U/mL; alpha amylase activity females: +38.61 U/mL; alpha amylase secretion males: +12.89 U/mL; alpha amylase secretion females: +13.4 U/mL	Significant increase in salivary alpha amylase activity (*p* = 0.001) and secretion rate (*p* = 0.023) in females but not males. Trend for higher levels of cortisol in females than males at rest (*p* −0.099) and during exercise (*p* = 0.070).	weak
Reference Ranges for Biomarkers										
Alpha-amylase: Concentration: Mean 92.4 U/mL Secretion rate (Euhydrated mean): 15.9 U/mL Activity (Euhydrated mean): 36.0 U/mL	Cortisol: 8:00 AM 140–690 nmol/L 4:00 PM 80–330 nmol/L Saliva: 11 pm-midnight < 0.248 ug/dL	Ghrelin: Total 520–700 pg/mL	Glucagon: 50–100 pg/mL	Insulin: 43–186 pmol/L (fasted)	Leptin: Males: 0.7–5.3 ng/mL Females: 3.3–18.3 ng/mL	PYY: (Euhydrated mean): 92.5 pg/mL	Testosterone: Men 10–35 nmol/L Women <3.5 nmol/L			

Abbreviations: OGTT = Oral glucose tolerance test, DH =Dehydration, NF = No Fluid, Iso = Isotonic saline, Hypo = Hypotonic saline, HYPO = Hypohydrated, RE = Rehydrated, RH = Relative Humidity, SAA = salivary alpha-amylase, BML = Body Mass Loss, W = Water, DHY = Dehydrated, LDAG = Low Dose Acute L-alanyl-L-Glutamine, HDAG = High Dose Acute L-alanyl-L-Glutamine, HHY – Hypohydrated, BL = Baseline, EUH = Euhydrated, HY50 = 5% Hypohydration, HY25 = 2.5% Hypohydration, Exercise DH = Exercise dehydration, Exercise HY = Exercise Hydrated, EH = Euhydrated + Heat, HH = Hypohydrated + Heat, EN = Euhydrated + Neutral, HN = Hypohydrated + Neutral. Carroll (2019)a = [83]; Carroll (2019)b = [30].

## Supplementary Materials

The following are available online at https://www.mdpi.com/2072-6643/12/9/2526/s1, File S1 PRISMA 2009 Checklist.
